# Clinical characteristics and outcomes in patients with *Anti*-MDA5 positive interstitial lung disease: A case series from a lung transplant center

**DOI:** 10.1016/j.rmcr.2025.102265

**Published:** 2025-07-22

**Authors:** René Hage, Eirini Chatzidaki, Christian F. Clarenbach, Macé M. Schuurmans

**Affiliations:** a Division of Pulmonology, University Hospital Zurich, 8091, Zurich, Switzerland; b Faculty of Medicine, University of Zurich, 8032, Zurich, Switzerland

**Keywords:** Autoimmune myopathy, Interstitial lung disease (ILD), Immunosuppressive therapy, Plasmapheresis, Fibrosis regression, Pulmonary function, Extracorporeal membrane oxygenation (ECMO)

## Abstract

**Background:**

Anti-Melanoma Differentiation-5 (MDA5)-positive dermatomyositis (DM) is a rare autoimmune disorder frequently complicated by rapidly progressive interstitial lung disease (RP-ILD), often with poor outcomes. Data from European tertiary care and transplant centers remain limited.

**Objective:**

To describe the clinical presentation, treatment course, and outcomes of five patients with *anti*-MDA5+DM-associated ILD at a lung transplant center.

**Methods:**

We retrospectively reviewed five patients with confirmed *anti*-MDA5 seropositivity and interstitial lung involvement between 2023 and 2025. Demographic data, clinical features, lung function, imaging, antibody levels, immunosuppressive treatment, and outcomes were analyzed.

**Results:**

Of the five patients 3 were female and two were male with a median age of 51 years (range 22–69). Clinical severity varied from mild cutaneous and pulmonary disease to fulminant RP-ILD. One patient required extracorporeal membrane oxygenation (ECMO) and eventually underwent lung transplantation. Treatment strategies included high-dose corticosteroids, rituximab, mycophenolate mofetil, tacrolimus, and intravenous immunoglobulins (IVIG). Patients with early initiation of rituximab or IVIG and declining *anti*-MDA5 antibody titers generally showed pulmonary stabilization. Serial lung function tests correlated with clinical improvement or deterioration. Cutaneous flare-ups persisted in some patients despite pulmonary stability.

**Conclusion:**

This case series underscores the heterogeneous trajectory of *anti*-MDA5+DM-ILD and highlights the importance of timely diagnosis, aggressive immunosuppressive treatment, and early transplant evaluation in severe cases. Serial monitoring of *anti*-MDA5 antibody titers and lung function testing may help guide therapeutic decisions and prognostication.

## 1 Introduction

Dermatomyositis (DM) is an idiopathic inflammatory myopathy characterized by immune-mediated inflammation of the skin and muscle. The identification of myositis-specific antibodies has refined the clinical classification of DM. Among these, anti-melanoma differentiation-associated gene 5 (MDA5) identifies a distinct clinical phenotype, often associated with clinically amyopathic dermatomyositis (CADM) and a predisposition to rapidly progressive interstitial lung disease (RP-ILD).

RP-ILD in the context of *anti*-MDA5 positivity has historically been associated with poor outcomes, although early combination immunosuppression has improved outcomes in selected cohorts [1]. The underlying immunopathology involves heightened type I interferon signaling, B cell activation, and immune complex-mediated alveolar injury [2]. Standard-of-care treatment typically includes high-dose corticosteroids combined with calcineurin inhibitors and/or cyclophosphamide [[3], [4], [5]]. Despite such regimens, disease progression to respiratory failure is not uncommon.

Lung transplantation is increasingly considered for selected patients with connective tissue disease-associated ILD, including those with *anti*-MDA5-positive RP-ILD [6,7]. However, data on post-transplant outcomes remain limited. We describe five cases of *anti*-MDA5-positive ILD followed at a tertiary university hospital setting, illustrating the clinical heterogeneity, treatment responses, and long-term outcomes, including one case of successful lung transplantation.

## 2 Methods

We conducted a retrospective case series of five patients diagnosed with *anti*-MDA5-positive ILD at the Division of Pulmonology, University Hospital Zurich, Switzerland, between January 2022 to May 2025. Inclusion criteria were: (1) serologic positivity for *anti*-MDA5 antibodies, (2) radiologic and/or histopathologic evidence of ILD, and (3) clinical follow-up of at least three months or until death or transplantation. Data were extracted from electronic medical records. Extracted data included demographics, clinical features, serologic profiles, radiologic and pulmonary findings, immunosuppressive treatments, and outcomes. *Anti*-MDA5 antibodies were measured via enzyme-linked immunosorbent assay (ELISA), with positivity defined as ≥5 %. Descriptive statistics were used to summarize the clinical characteristics. Written informed consent was obtained from all patients. Baseline characteristics are shown in Table 1.

**Table 1:** Baseline characteristics.

Pt	Age/Sex	Antibodies	ILD Severity	Key Treatments	Lung Tx	Latest FVC	DLCO	Current Status
1	22/F	MDA5+	ARDS	RTX, Tac, IVIG, Endoxan, PEX	No	59 %	72 %	Stable
2	51/M	MDA5+	ARDS	RTX, MMF, IVIG, Tac	Yes	FEV1 3.7L	53 %	Stable post-transplant
3	69/M	MDA5+	RP-ILD	TOCI, MMF, RTX, IVIG	No	54 % (stable)	53 % (stable)	Controlled disease
4	54/F	MDA5+	Mild ILD	MMF, RTX, IVIG	No	94 %	76 %	Good control
5	53/F	MDA5+	Moderate ILD	RTX, Tac, Steroids	No	102 %	85 %	Stable, fibromyalgia symptoms

**Abbreviations:** ARDS: acute respiratory distress syndrome; DLCO: diffusion capacity for carbon monoxide; Endoxan: cyclophosphamide; F: female; FEV1: forced expiratory volume in 1 second; FVC: forced vital capacity; ILD: interstitial lung disease; IVIG: intravenous immunoglobulins; M: male; MDA5: melanoma differentiation-associated gene 5; MMF: mycophenolate mofetil; PEX: plasmapheresis; Pt: patient; Ro52: *anti*-Ro52 antibody; RP-ILD: rapidly progressive interstitial lung disease; RTX: rituximab; Tac: tacrolimus; TOCI: tocilizumab; Tx: transplantation.

## 3 Case descriptions

### 3.1 Case 1

#### 3.1.1 Initial presentation

A 22-year-old previously healthy woman of Indian origin presented in November 2022 with acute respiratory failure shortly after returning from India. During her travel, she had developed fever, dry cough, and joint pain, which progressed to exertional dyspnea, low-grade fever, and symmetric arthritis of the wrists and fingers.

#### 3.1.2 Diagnostic findings

**Fig. 1:**
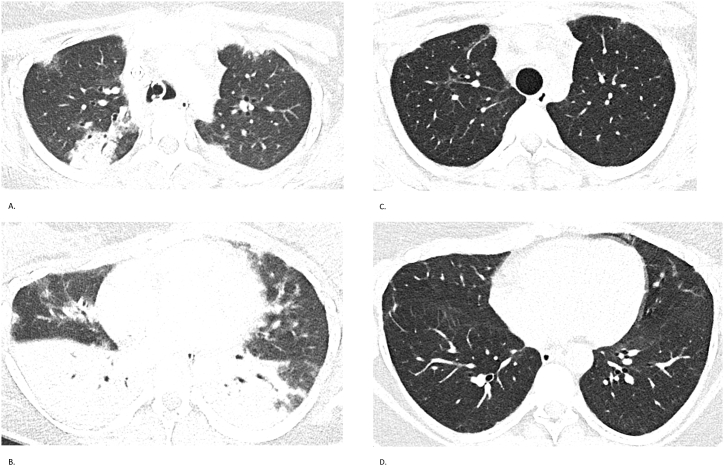
Case 1 – Chest CT findings Serial chest CTs of a 22-year-old woman with *anti*-MDA5-positive ILD. (A, B) CT on November 25, 2022, shows extensive bilateral ground-glass opacities and consolidations. (C, D) CT on May 28, 2024, demonstrates marked radiological improvement with resolution of acute inflammatory changes and residual subpleural fibrosis.

High-resolution CT of the chest revealed bilateral ground-glass opacities and consolidations with rapidly progressive interstitial lung disease (Fig. 1). Serology was notable for elevated titers of *anti*-MDA5 antibodies, with a peak *anti*-MDA5 IgG index of 21 %, declining to 11 % and eventually becoming undetectable after eight months. Initial spirometry in April 2023 demonstrated a severe restrictive ventilatory defect: Forced Vital Capacity (FVC) 1.45 L (45 %). Gradual improvement was observed, reaching FVC 2.05 L (63 %) by September 2024.

#### 3.1.3 Treatment course

Immunosuppressive therapy included high-dose corticosteroids, pulsed intravenous methylprednisolone, intravenous cyclophosphamide (800 mg), rituximab ((1000 mg loading, followed by 500 mg every six months), intravenous immunoglobulins (IVIG) (40 g/month), tacrolimus (target trough 7–9 ng/mL, later reduced to 5–7 ng/mL), and five sessions of plasmapheresis.

#### 3.1.4 Clinical course and outcome

The patient developed refractory hypoxemia requiring veno-venous extracorporeal membrane oxygenation (vv-ECMO) for 47 days, in combination with mechanical ventilation and prone positioning. The ICU stay was complicated by critical illness neuropathy, device-associated thrombosis near the atrial septum, recurrent ventilator-associated pneumonia due to multidrug-resistant *Pseudomonas* and *Klebsiella*, cytomegalovirus (CMV) reactivation, decubitus ulcers, paralytic ileus, and transient liver enzyme elevations. Extensive microbiological testing was negative for tuberculosis, hepatitis viruses, HIV, and endemic pathogens. Following ICU discharge, the patient's lung function gradually improved. Although initially evaluated and listed for lung transplantation, her clinical and pulmonary status stabilized significantly, and lung transplant was deferred.

### 3.2 Case 2

#### 3.2.1 Initial presentation

A 51-year-old man presented in late October 2022 with fever, dry cough, progressive dyspnea, fatigue, significant weight loss (∼15 kg), and arthralgia. These symptoms had gradually worsened over several months, beginning after a trip to Thailand and a SARS-CoV-2 infection in March 2022. He had received a COVID-19 booster vaccine in October 2022. Upon admission, he rapidly deteriorated and required intubation, followed by vv-ECMO support initiated in November 2022.

#### 3.2.2 Diagnostic findings

**Fig. 2:**
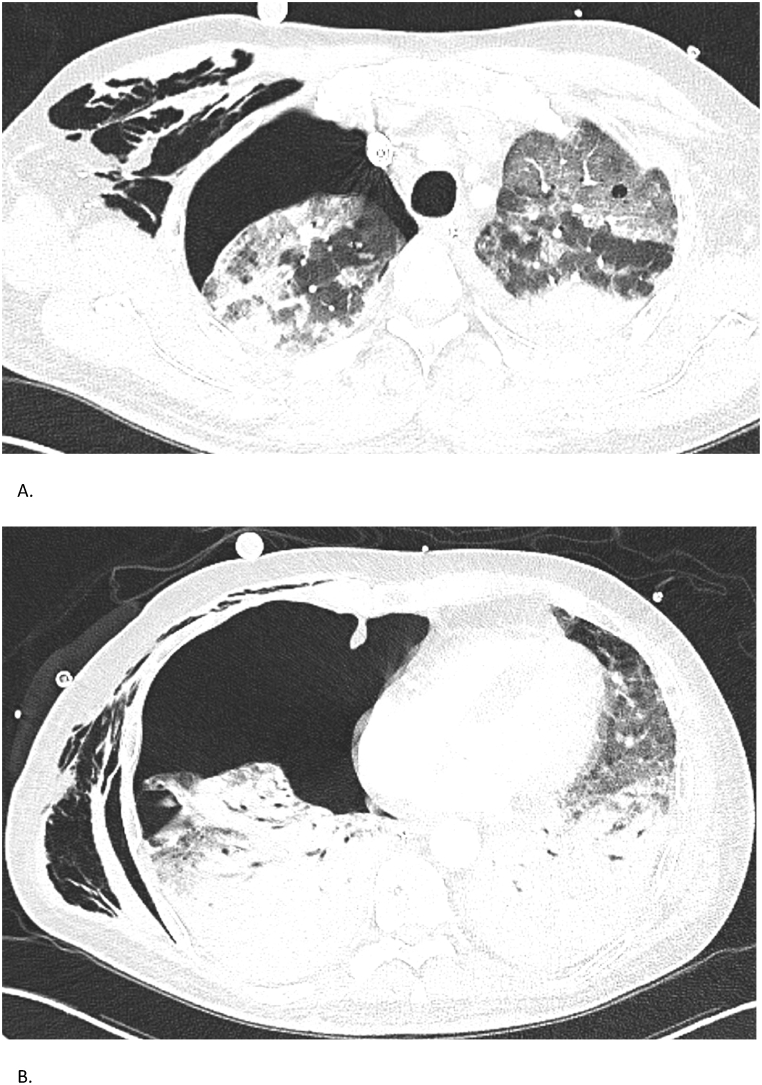
Case 2 – Chest CT findings Chest CTs of a 51-year-old man with *anti*-MDA5-associated RP-ILD. (A, B) CT on November 10, 2022, reveals diffuse fibrotic changes with honeycombing and right-sided pneumothorax. Despite chest drainage, the lung shows incomplete re-expansion, consistent with trapped lung.

High-resolution chest CT revealed fibrotic interstitial lung disease with honeycombing and bronchiectasis, mainly affecting the lower lobes and right middle lobe. Shortly after ICU admission, the patient developed a right-sided pneumothorax, which was managed with chest tube drainage; however, the lung failed to re-expand, likely due to underlying fibrosis and alveolar damage (Fig. 2). Serologic testing showed markedly elevated *anti*-MDA5 IgG levels (72 %), which gradually declined to 8 % after initiation of immunosuppressive treatment.

#### 3.2.3 Treatment course

The patient received pulsed intravenous methylprednisolone (250 mg/day for 3 days), intravenous cyclophosphamide (1435 mg), ciclosporin, and plasmapheresis (11 sessions). Due to refractory acute respiratory distress syndrome (ARDS, with rapid onset, so that personal informed consent could not obtained), he underwent emergency bilateral lung transplantation in December 2022.

#### 3.2.4 Clinical course and outcome

The postoperative course in the ICU was complicated by prolonged ventilator dependence, critical illness polyneuropathy with flaccid tetraparesis, dialysis-requiring acute kidney injury, and infections complications including Epstein-Barr Virus (EBV) and CMV reactivation. They were managed with targeted antimicrobial therapy and adjustment of immunosuppression. Maintenance immunosuppressive therapy included tacrolimus (target trough-adjusted to kidney function), mycophenolate mofetil (MMF), prednisone, and IVIG. *Anti*-MDA5 antibodies remained persistently undetectable post-transplant. Lung function stabilized with FEV1 3.70 L (95 %), and the patient returned to work at 80 % capacity in an office setting. Written informed consent for publication was obtained.

### 3.3 Case 3

#### 3.3.1 Initial presentation

A 69-year-old man presented in April 2023 with *anti*-MDA5 positive dermatomyositis. Symptom onset was traced back to February 2023 during a stay in Thailand. He reported rapidly progressive exertional dyspnea (NYHA class IV), dry cough, heliotrope rash, Gottron's papules, and mild proximal muscle weakness.

#### 3.3.2 Diagnostic findings

**Fig. 3:**
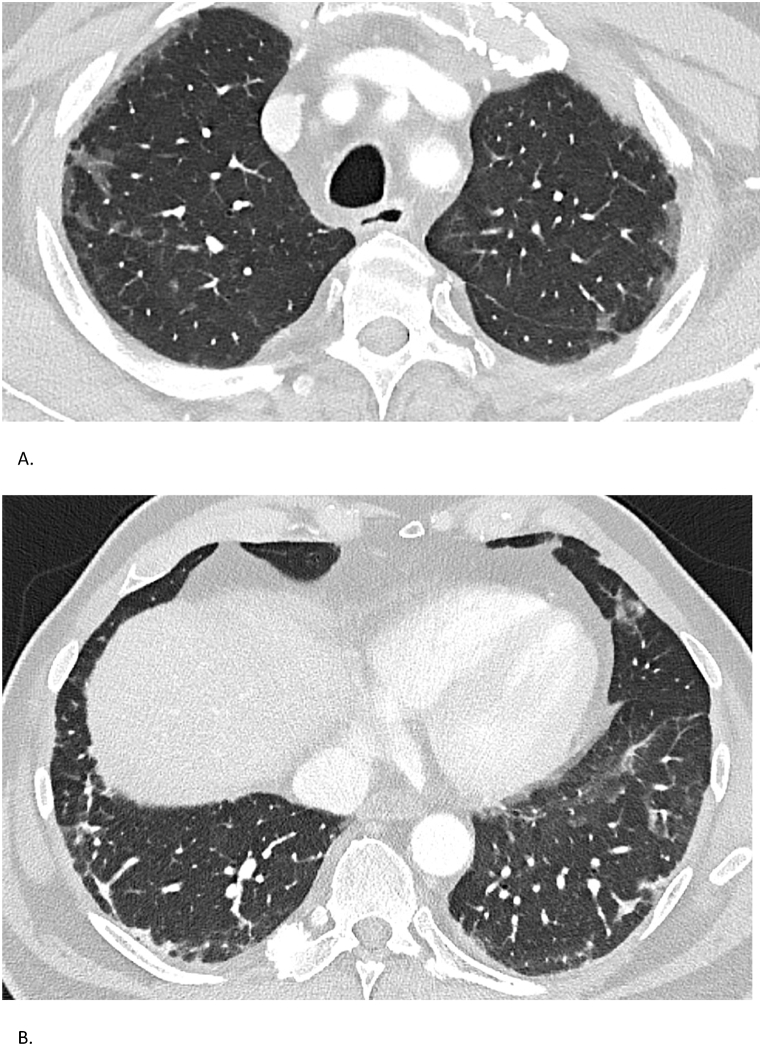
Case 3 – Chest CT findings Serial chest CTs of a 69-year-old man with *anti*-MDA5-positive ILD. (A) CT on May 3, 2023, displays subpleural fibrosis and diffuse ground-glass opacities. (B) CT on the same date in a different plane confirms extensive fibrotic changes without honeycombing.

High-resolution chest CT demonstrated extensive subpleural fibrosis and ground-glass opacities, without honeycombing (Fig. 3). Lung function revealed a restrictive pattern with FVC of 2.73 L (68 %) and diffusing capacity (DLCO) of 69 % in April 2023. *Anti*-MDA5 IgG titers were initially 40 % and became undetectable on treatment by October 2023.

#### 3.3.3 Treatment course

Immunosuppressive therapy included pulsed high-dose corticosteroids, MMF (3g/day), intravenous cyclophosphamide (600 mg/m^2^), monthly IVIG, and tocilizumab (8 mg/kg every 4 weeks) and five cycles of plasmapheresis. Despite aggressive therapy, the patient initially experienced functional decline and oxygen requirement, with FVC falling to 2.14 L (54 %) and DLCO to 53 %.

#### 3.3.4 Clinical course and outcome

From late 2023 onward, the patient experienced steady clinical and functional improvement. By January 2025, FVC improved to 3.53 L (91 %) and DLCO to 97 %. Follow-up CT conformed regression of consolidations and persistent but stable subpleural bands. The patient currently remains on hydrocortisone, MMF (2 g/day), and tocilizumab every 6 weeks. He reports minimal exertional dyspnea (NYHA class I) and stable overall disease. Written informed consent for publication was obtained.

### 3.4 Case 4

#### 3.4.1 Initial presentation

A 54-year-old woman was diagnosed with *anti*-MDA5-positive dermatomyositis (DM) in July 2023. She presented with bilateral wrist arthritis, exertional dyspnea, dry cough, Gottron's papules, periungual ulcerations, Raynaud's phenomenon, and a pericardial effusion confirmed by echocardiography.

#### 3.4.2 Diagnostic findings

**Fig. 4:**
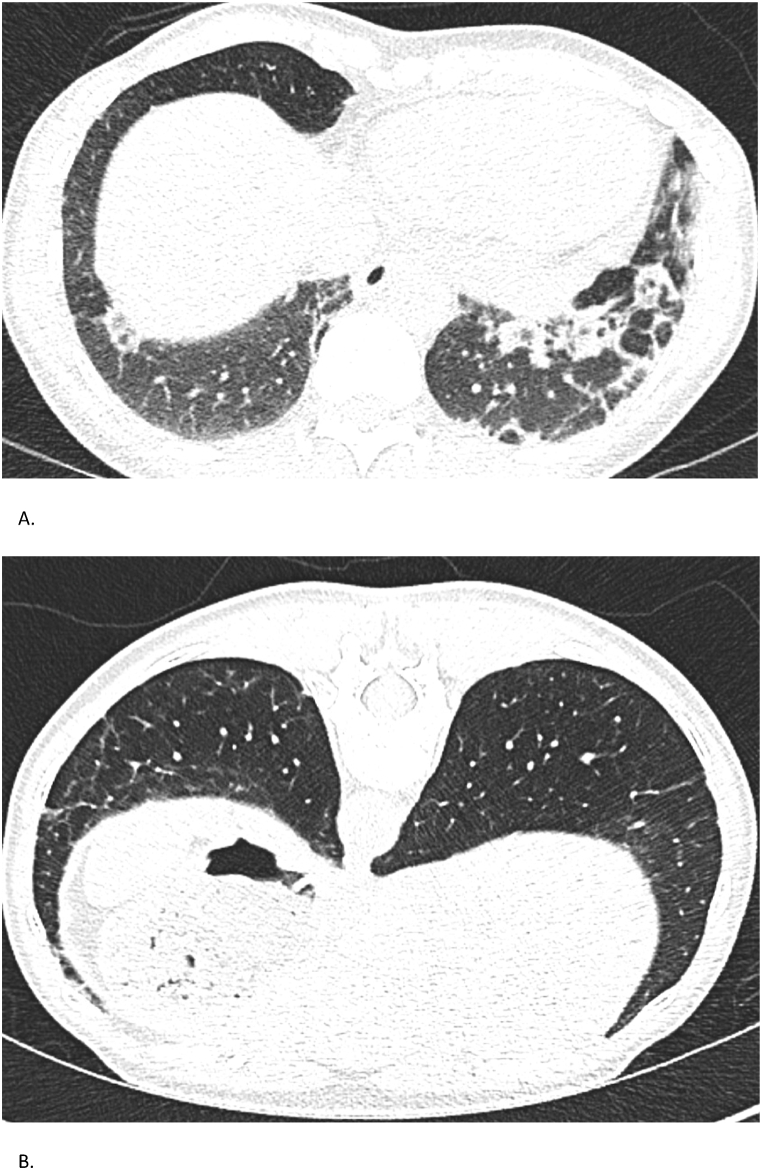
Case 4 – Chest CT findings Serial chest CTs of a 54-year-old woman with *anti*-MDA5 positive dermatomyositis. (A) CT on September 15, 2023, shows early fibrotic changes and subpleural consolidations. (B) CT on February 14, 2025, demonstrates partial regression of prior abnormalities with new subtle reticulations.

Serologic testing revealed a high *anti*-MDA5 IgG titer (73 %), decreasing to 30 % by February 2024 and undetectable by January 2025. Myositis- and systemic sclerosis-associated antibodies were negative. Complement levels were normal and cryoglobulins absent. High-resolution CT showed bilateral lower lobe-predominant reticular opacities, subpleural consolidations, and early fibrotic changes (Fig. 4). Lung function showed a restrictive pattern with FVC 3.20 L (84 %) and DLCO of 61 % in June 2023, improving to FVC 3.48 L (93 %) and DLCO 73 % in April 2025.

#### 3.4.3 Treatment course

Initial therapy included high-dose corticosteroids (tapered and stopped by May 2024), and azathioprine (discontinued due to inefficacy). MMF was initiated in January 2024. Intermittent IVIG (Octagam) was used to control dermal flares with good effect. Rituximab was started in August 2023 and continued every 6 months.

#### 3.4.4 Clinical course and outcome

Despite evolving CT findings with alternating regressing and emerging consolidations, pulmonary function improved steadily. The 6-min walk distance increased from 600 to 712 m, with reduced dyspnea. *Anti*-MDA5 antibodies became undetectable within one year. Clinically, she reported stable respiratory symptoms (NYHA class I), mild Raynaud's syndrome, and only intermittent skin activity. No infections or hospitalizations occurred. Her disease remains well-controlled. Written informed consent for publication was obtained.

### 3.5 Case 5

#### 3.5.1 Initial presentation

A 53-year-old woman was diagnosed with *anti*-MDA5-positive dermatomyositis (DM) with interstitial lung disease (ILD) in August 2023. She had developed progressive exertional dyspnea, symmetric arthralgia (MCP joints), Gottron's papules, heliotrope rash, mechanic's hands, oral aphthae, and photosensitivity. Raynaud's phenomenon was absent at initial presentation.

#### 3.5.2 Diagnostic findings

**Fig. 5:**
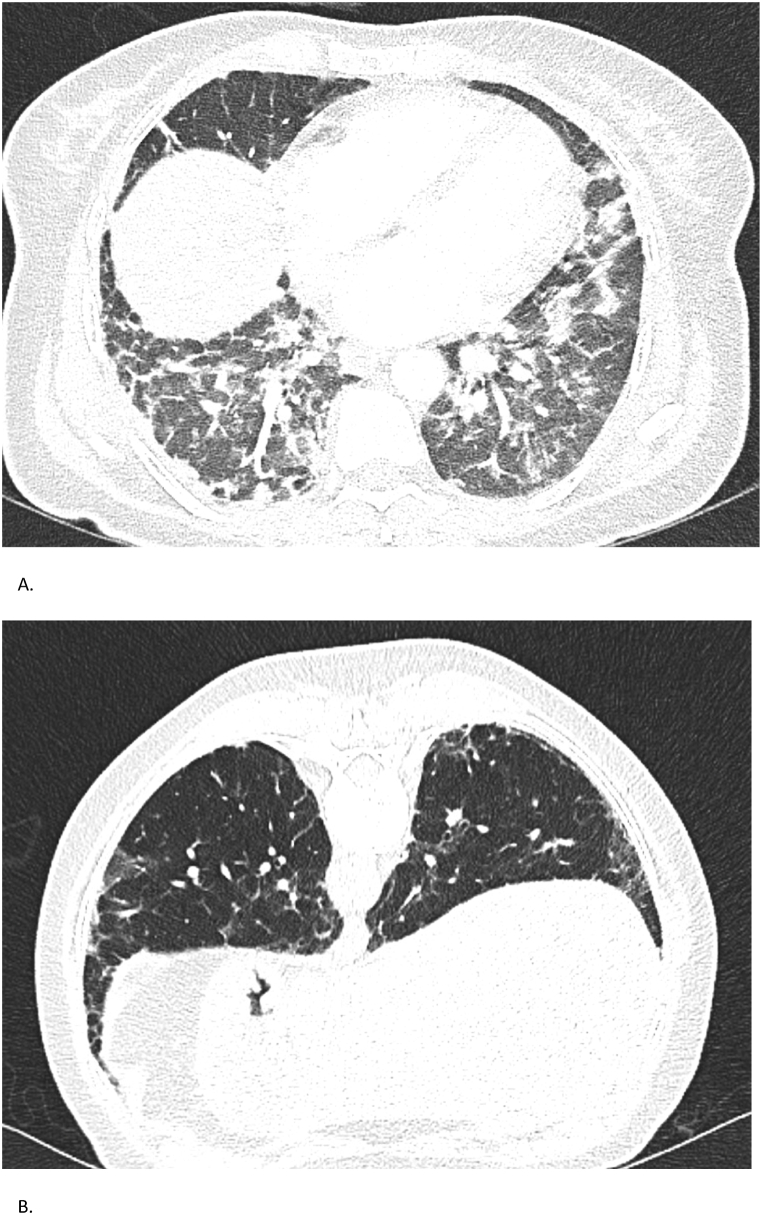
Case 5 – Chest CT Findings Serial chest CTs of a 53-year-old woman with MDA5-positive ILD. (A) CT on August 3, 2023, reveals widespread ground-glass opacities and consolidations with early fibrosis. (B) CT on April 15, 2025, shows significant resolution with residual subpleural fibrotic changes, estimated at 10–20 %.

Chest CT in August 2023 showed bilateral ground-glass opacities, consolidations, and subpleural reticulation (Fig. 5). Lung function tests revealed a restrictive pattern with FVC 2.07L (73 %) and severely impaired DLCO (45 %). By April 2025, FVC had improved to 2.27L (82 %) and DLCO to 93 %, with CT showing resolution of inflammatory infiltrates and only 10–20 % subpleural fibrosis. Serology showed *anti*-MDA5 IgG 76 %, an ANA titer of 1:320 (AC-4 pattern), and normal CK and myoglobin. Myositis-specific antibodies, RF, and *anti*-CCP were negative. Muscle MRI showed inflammation in deltoids, biceps, and gluteal muscles. MMT8 scored 62/80, indicating mild proximal weakness; Frailty Index FI-2 was inconclusive due to language barriers. Skin biopsy revealed interface dermatitis. Cardiac evaluations were unremarkable. Tumor screening and infection work-up were negative.

#### 3.5.3 Treatment course

Initial therapy included prednisone 50 mg/day (tapered to 6.25 mg by April 2025), tacrolimus (target trough 7–9 ng/mL), and rituximab (2 × 1 g in October 2023). Maintenance rituximab was administered based on CD19/CD20 status every 6 months (500 mg). No additional DMARDs or IVIG were required.

#### 3.5.4 Clinical course and outcome

Dyspnea improved steadily, with a 6-min walk test of 450 m in April 2025 without desaturation. *Anti*-MDA5 became undetectable. CT imaging confirmed mild residual fibrosis without honeycombing or active alveolitis. Despite good systemic control, she developed diffuse myalgia without objective weakness, consistent with fibromyalgia. In April 2025, she also developed Raynaud's phenomenon; repeat nailfold capillaroscopy showed microangiopathy, though no ulcers occurred. She remains on low-dose prednisone, tacrolimus, and rituximab, with stable pulmonary and systemic disease. Fibromyalgic symptoms are managed conservatively. A phased return to work was initiated (50 % in April 2025, aiming to increase workload over time). Written informed consent for publication was obtained.

## 4 Discussion

This case series highlights the remarkable clinical heterogeneity and therapeutic challenges associated with *anti*-MDA5-positive interstitial lung disease (ILD). Despite a common serologic marker, our five patients demonstrated widely variable trajectories, ranging from mild subacute ILD with practically preserved pulmonary function to fulminant respiratory failure requiring ECMO support and (emergency) lung transplantation. Although the pathogenesis of *anti*-MDA5-positive ILD remains incompletely understood, infectious triggers—including SARS-CoV-2—and, in rare cases, immune activation following COVID-19 vaccination have been suggested as potential initiating events [8]. In our series, one patient developed symptoms following a respiratory illness acquired abroad, and another reported symptom onset shortly after COVID-19 vaccination, suggesting possible post-infectious or post-vaccinal immune activation.

Early diagnosis and prompt initiation of immunosuppressive therapy remain critical to improving outcomes. Despite a lack of large, randomized trials, combination immunosuppressive therapy is widely adopted as standard or care. Standard first-line treatment includes high-dose glucocorticoids, calcineurin inhibitors, with or without intravenous cyclophosphamide [5].

Japanese studies have advocated for upfront triple immunosuppressive therapy, showing improved 6-month survival compared to step-up immunosuppression [3]. However, this benefit is not consistently observed in Western cohorts. For example, a large retrospective study by Gono et al., found no clear survival advantage of triple therapy over dual or monotherapy [9], suggesting that geographic and immunogenetic factors, including epitope specificity, may influence treatment response. One potential explanation lies in immunogenetic and epitope-specific variability [10]. A comparative study of Japanese and North American patients revealed divergent B-cell epitope recognition: Japanese patients primarily developed antibodies against MDA5 fragments A, B, and E, particularly fragment A, which correlated with RP-ILD and mortality, whereas North American patients targeted fragment H^10^. These geographic patterns may reflect distinct environmental exposures or genetic backgrounds and could influence clinical course and treatment efficacy. As such, results from Japanese cohorts, including those supporting triple therapy, may not be directly generalizable to European or North American populations.

Alternative agents such as Janus kinase (JAK) inhibitors (e.g., tofacitinib) and interleukin-6 (e.g., tocilizumab) are increasingly used in refractory cases [11]. Small observational studies suggest clinical stabilization or improvement, particularly when introduced early [11]. Nonetheless, robust prospective data are lacking.

Second line options for refractory cases include rituximab [5], mycophenolate mofetil [5], tofacitinib [2,12], plasmapheresis [13], or intravenous infusion of human umbilical cord mesenchymal stem cells (HUC-MSCs) [14] have shown promise in case series and early-phase studies but remain unvalidated in larger cohorts [3]. One patient in our series (Case 3) was treated with tocilizumab as a steroid-sparing agent with apparent disease stabilization. In three of our patients (Case 1–3), plasmapheresis was used successfully in refractory disease, aligning with recent meta-analytic data suggesting improved one-year survival when used early in RP-ILD [13,15].

Plasmapheresis is increasingly being recognized as a rescue therapy for MDA5+DM-ILD patients with rapidly progressive disease refractory to standard treatment. A recent systematic review and meta-analysis including 148 patients across six cohort studies demonstrated a significantly improved one-year survival in patients treated with plasmapheresis in addition to immunosuppressive therapy [15]. Survival benefit was greatest when plasmapheresis was initiated early, ideally within 2 weeks of diagnosis. In addition to survival, some studies reported trends towards improved pulmonary function (forced vital capacity, FVC, and diffusing capacity for carbon monoxide, DLCO), radiographic resolution, and reduced levels of serum biomarkers such as Krebs von den Lungen (KL-)6 and ferritin [15]. These findings support considering plasmapheresis in patients with refractory or fulminant RP-ILD, particularly when standard immunosuppression is limited by infection or toxicity. However, further prospective studies are needed to define optimal timing and patient selection.

One patient in our series (Case 2) underwent bilateral lung transplantation after refractory ARDS requiring ECMO, with excellent functional recovery and persistent seronegativity for *anti*-MDA5 antibodies. Lung transplantation is a life-saving option in medically refractory cases, emphasizing the importance of timely referral to lung transplant centers. ECMO can serve as a bridge to recovery or transplantation in selected patients. A recent series by Lian et al. described two *anti*-MDA5+ ILD patients bridged with ECMO [6]. One patient recovered fully with antibody clearance, while the other died from early recurrence of disease with persistently high *anti*-MDA5 titers. Among 11 reported transplant recipients, 90 % survived without recurrence [6]. These findings suggest that antibody clearance may reflect improved prognosis, whereas persistently high titers may predict relapse risk.

Classification challenges further complicate early recognition and management. Although MDA5+DM-ILD is now recognized as a distinct clinical entity, up to 30 % of patients may not meet the 2017 EULAR/ACR criteria for idiopathic inflammatory myopathies (IIM) [16]. Many are classified as having interstitial pneumonia with autoimmune features (IPAF) [17], a heterogeneous research category lacking treatment guidelines. This can lead to undertreatment of high-risk patients who otherwise resemble classical MDA5+DM-ILD in clinical severity and prognosis. Recent proposals to revise classification criteria, such as replacing *anti*-Jo-1 with *anti*-MDA5 antibodies and including ILD as a core domain, may improve sensitivity [16].

However, expanding classification criteria also carries the risk of overdiagnosis. IPAF encompasses a broad phenotype spectrum and misclassifying less aggressive or unrelated ILD phenotypes as MDA5+ could expose patients unnecessarily aggressive immunosuppression. One study emphasized this concern, showing that among patients initially labeled as IPAF, many did not show MDA5 seropositivity or progressive disease [18]. Ultimately, any revision of the classification framework must balance timely recognition of high-risk subtypes with diagnostic specificity.

Some of these cases likely reflected incidental serological findings, particularly in the presence of other autoantibodies or confounding conditions. This underscores the importance of interpreting MDA5 seropositivity in the appropriate clinical context. While expanded criteria may enhance sensitivity, they must be balanced against the risk of overdiagnosis and unnecessary treatment in seropositive individuals without clear clinical correlates [18].

In conclusion, this case series shows that *anti*-MDA5-positive ILD is a rare but severe autoimmune lung disease that demands early recognition, aggressive and multimodal immunosuppression, and multidisciplinary coordination. Lung transplantation is feasible and effective in carefully selected patients, particularly when antibody titers are declining pre-transplant. Future research should aim to refine diagnostic criteria, validate dynamic biomarkers, and optimize immunosuppressive strategies based on individual risk profiles. Our findings support integrating *anti*-MDA5 serology and early transplant evaluation into routine management of high-risk ILD phenotypes.

## CRediT authorship contribution statement

**René Hage:** Conceptualization, Data curation, Formal analysis, Investigation, Methodology, Writing – original draft, Writing – review & editing. **Eirini Chatzidaki:** Formal analysis, Investigation, Methodology, Writing – original draft, Writing – review & editing. **Christian F. Clarenbach:** Formal analysis, Investigation, Methodology, Writing – original draft, Writing – review & editing. **Macé M. Schuurmans:** Data curation, Formal analysis, Methodology, Supervision, Writing – original draft, Writing – review & editing.

## Declaration of generative AI and AI-assisted technologies in the writing process

During the preparation of this work the authors used ChatGPT (OpenAI, San Francisco, USA) to improve the readability and clarity of the manuscript. After using this tool, the authors reviewed and edited the content as needed and take full responsibility for the content of the published article.

## Funding statement

This research did not receive any specific grant from funding agencies in the public, commercial, or not-for-profit sectors.

## Declaration of competing interest

The authors declare that they have no known competing financial interests or personal relationships that could have appeared to influence the work reported in this paper.
